# Physiological assessment of the effects of changing water levels associated with reservoir management on fattening rates of neotropical migrants at a stopover site

**DOI:** 10.1093/conphys/cou017

**Published:** 2014-05-15

**Authors:** D. N. Wagner, D. J. Green, M. Pavlik, J. Cooper, T. D. Williams

**Affiliations:** 1 Centre for Wildlife Ecology, Department of Biological Sciences, Simon Fraser University, 8888 University Drive, Burnaby, BC, Canada V5A 1S6; 2 Cooper Beauchesne and Associates Ltd, Kootenay Office, 3308-1123 West 2nd Street, Box 2508, Revelstoke, BC, Canada V0E 2S0; 3 Cooper Beauchesne and Associates Ltd, Head Office, Box 646, Errington, BC, Canada V0R 1V0

**Keywords:** Fattening rate, migratory birds, reservoir operations, riparian habitat

## Abstract

Riparian habitat makes up a small fraction of the landscape but provides important stopover habitat for migratory birds. Hydroelectric dam operations cause fluctuations in water levels that can change the amount or quality of riparian habitat, which in turn might affect potential fattening rates of migrant birds. Here we used plasma metabolite analysis to estimate variation in fattening rate in relationship to variable water levels associated with reservoir management in four species of neotropical migratory songbirds using riparian habitat at a dam-impacted stopover site in Revelstoke, British Columbia, Canada. Residual plasma triglyceride, our measure of estimated fattening rate, varied systematically with time of day and Julian date and varied consistently among species, but did not vary with age or sex. Controlling for potentially confounding variables, we found no inter-annual variation in estimated fattening rate, even though there were marked differences in water levels among years. Likewise, there was no relationship between daily variation in water levels and estimated fattening rate. Data on feather isotopes (δD), indicative of migratory origin, did not add explanatory power to our models. There was inter-annual variation in plasma glycerol and β-hydroxybutyrate levels and significant, though weak, relationships between these metabolites and water level (higher metabolite levels when drier) that might indicate effects on ‘body condition’ independent of fattening rate. Our study suggests that, at present, although hydroelectric dam operations influence water levels in the Arrows Lake Reservoir and adjacent riparian habitats, this does not significantly impact fattening rates of migratory passerines using these habitats.

## Abstract (precis)

We used plasma metabolite analysis to estimate variation in fattening rate in four neotropical migratory songbirds utilising riparian habitat at a dam-impacted stopover in British Columbia, Canada. Our study suggests that although hydroelectric dam operations influence water levels this does not significantly impact fattening rates of birds using these habitats.

## Introduction

Conservation of migratory birds requires research across the entire avian annual cycle to inform potential management and habitat conservation (Faaborg *et al.*, 2010). For many songbirds, resources in lowland, riparian habitats are considered to be especially important for stopovers during migration, particularly in the western USA and Canada (Skagen *et al.*, 1998; Wiebe and Martin, 1998; Finch and Yong, 2000). Riparian habitat makes up a small fraction (∼1%) of the western USA landscape (Knopf *et al.*, 1988) and this fraction continues to decline as natural flow regimens are modified, floodplains are developed and land is cleared for agriculture or urban development (Poff *et al.*, 1997). The continued loss and degradation of this apparently critical stopover habitat may contribute to future population declines of western songbirds (Ohmart, 1994; Skagen *et al.*, 2005). Indeed, it has been proposed that preservation of stopover habitat is crucial to the conservation of migratory songbirds (Hedenström and Alerstam, 1998; Petit, 2000; Skagen *et al.*, 2005; Martin *et al.*, 2007; Carlisle *et al.*, 2009).

Hydroelectric development has contributed to the loss of riparian habitat in the Pacific Northwest; 16 major dams have created a reservoir system that has led to the loss of >87% of the riparian habitat within the Canadian portion of the Columbia River Basin (Moody *et al.*, 2007; Utzig and Schmidt, 2011). Hydroelectric dam operations that lead to fluctuations in water levels of reservoirs can continue to flood remnant riparian habitat used by migrant birds in the summer and autumn such that water levels can vary throughout the migration season, as well as from year to year (Utzig and Schmidt, 2011). Higher water levels reduce the amount of riparian habitat available to migrants because riparian habitat located within the drawdown zone of the reservoir is flooded and unavailable (Green *et al.*, 2011). Furthermore, increasing densities of foraging birds in a smaller area of habitat could increase competition, and these effects could combine to reduce potential fattening rates (but see Green *et al.*, 2011). Alternatively, higher water levels, particularly earlier in the migration season, might increase plant growth and insect productivity in riparian habitat, which could increase fattening rates of migrants. Thus, inter-annual variation in daily water levels and seasonal variation in water levels might cause systematic seasonal or inter-annual variation in fattening rates of migrant birds using the riparian habitat.

Several recent studies have investigated the impact dams might have on local and migrant avifauna using the riparian habitats in the drawdown zones (Green *et al.*, 2011; Quinlan and Green, 2012; Cooper Beauchesne and Associates Ltd, 2013). Green *et al.* (2011) measured the rate of mass gain by regression on capture time of migrants during stopover and concluded that mass gain did not vary annually or with date, and that it was not influenced by annual or weekly variation in reservoir water levels. However, although several investigators have used daily mass gain by migratory songbirds to assess and compare stopover habitat quality (e.g. Dunn, 2000, 2002; Ktitorov *et al.*, 2008), other studies have shown that the use of ‘static’ measures of body condition (e.g. mass or fat score) at the time of capture does not provide meaningful information on fattening rates (Guglielmo *et al.*, 2002, 2005; Williams *et al.*, 2007; Evans Ogden *et al.*, 2013).

Here we take a physiological approach, estimating the fattening rates of neotropical migrants at a stopover site using plasma metabolite analysis. Plasma metabolite analysis uses residual plasma triglyceride levels (controlling for body mass and other covariates) to estimate rates of fattening or refuelling; high levels of plasma triglyceride represent high fattening rates (Jenni-Eiermann and Jenni, 1994; Guglielmo *et al.*, 2005; Williams *et al.*, 2007; Smith and McWilliams, 2010; Seewagen *et al.*, 2011). Other studies have typically also measured other metabolites associated with fasting or mass loss (glycerol, β-hydroxybutyrate), as we do here, but residual plasma triglyceride level is more informative for estimation of fattening rate and for detection of site differences than is variation in glycerol and β-hydroxybutyrate (e.g. Guglielmo *et al.*, 2002; Acevado Seaman *et al.*, 2006; Williams *et al.*, 2007; Evans Ogden *et al.*, 2013). Plasma metabolite analysis has been validated for the estimation of relative fattening rates, in both captive (Seaman *et al.*, 2005; Cerasale and Guglielmo, 2006) and free-living birds (Schaub and Jenni, 2001; Guglielmo *et al.*, 2005; Anteau and Afton, 2008). Thus, plasma triglyceride tends to be the single most useful lipid metabolite for assessment of fattening and fuel deposition in free-living migratory birds (Smith and McWilliams, 2010).

We used plasma metabolite analysis to estimate the variation in fattening rate in relationship to variable water levels associated with reservoir management in four species of neotropical migratory songbirds [Common Yellowthroat, *Geothlypis trichas* (COYE); Orange-crowned Warbler, *Oreothlypis celata* (OCWA); Wilson's Warbler, *Cardellina pusilla* (WIWA); and Yellow Warbler, *Setophaga petechia* (YWAR)] using riparian habitat at a dam-impacted stopover site in Revelstoke, British Columbia. The specific goals of this study were as follows: (i) to quantify water levels at our study site and to demonstrate marked variation in water levels, both among and within years, associated with reservoir operations; (ii) to confirm systematic relationships between residual plasma triglyceride, time of day and Julian date consistent with this, providing an index of estimated fattening rate; (iii) to determine whether residual plasma triglyceride (estimated fattening rate) and glycerol or β-hydroxybutyrate (putative ‘condition’ measures; see Discussion) vary annually, reflective of inter-annual variation in water levels, and with feather stable-hydrogen-isotope values, reflecting the geographical origin (breeding latitude) of migratory birds (Hobson and Wassenaar, 2008); (iv) to investigate species differences in estimated fattening rate; and (v) to test the effect of daily variation in water level (cf. ‘year’) on estimated fattening rate directly.

## Materials and methods

### Fieldwork and study site

**Figure 1:**
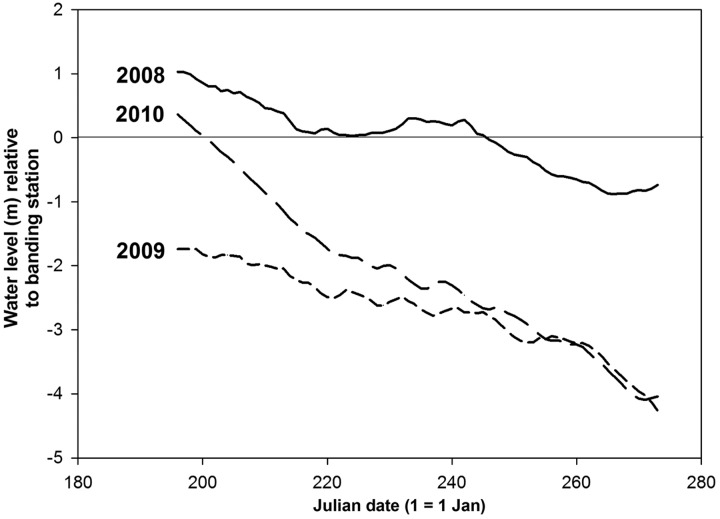
Variation in water levels among years and during the banding period for 2008–2010 at Machete Island, located in the drawdown zone of Arrow Lakes Reservoir, Revelstoke, BC, Canada. Water level = current reservoir elevation minus elevation of the banding station (438.7 m) minus), such that positive values indicate that the site is inundated by water.

Fieldwork was conducted at the Columbia River Revelstoke Migration Monitoring Station, Machete Island, ∼2 km south of Revelstoke, British Columbia, Canada (50°58′13.29″N; 118°11′56.14″W) from 15 July to 30 September 2008, 2009 and 2010. Machete Island is a semi-wooded riparian habitat of about 30 ha, composed of deciduous forest dominated by cottonwood (*Populus* spp.) with a diverse understory, surrounded by willow scrub, surrounded by shrub-savannah, grasslands, wetlands and the Columbia river (Green *et al.*, 2011). We sampled an area of about 1.7 ha of the ∼15 ha riparian forest of Machete Island, which is representative of the ∼144 ha of riparian forest habitat in the entire Revelstoke Reach (below 441 m). This is one of the few remaining pieces of riparian habitat within the Canadian portion of the Columbia River Basin. Machete Island is located in the drawdown zone of Arrow Lakes Reservoir (defined as the footprint of the reservoir when the water level is the maximum allowed, i.e. 440 m), which undergoes annual fluctuation in water level due to dam operations at BC Hydro's upstream Revelstoke and Mica Dams and downstream Keenleyside Dam. The lowest water levels at Machete Island tend to occur in late winter/early spring, and then rise with snowmelt in May. Full pool, if reached, usually occurs in early summer, followed by a gradual decline in water level throughout the late summer and autumn, although this pattern varies annually (Fig. 1). The variation in water levels associated with reservoir operations and the associated flooding can reduce the available riparian habitat by 40–90% at our study site (Green *et al.*, 2011).

Common Yellowthroat, Yellow Warbler, Orange-crowned Warbler and Wilson's Warbler are small neotropical migratory passerines that breed from east to west in North America, including western Canada and Alaska, and over-winter in Mexico and Central America. These species forage primarily in shrubs and understory trees and may be impacted most by changing water levels (Green *et al.*, 2011).

### Capture and blood sampling

We captured and blood sampled 107 COYE, 76 YWAR, 62 OCWA and 51 WIWA in 2008; 165 COYE, 87 YWAR, 102 OCWA and 43 WIWA in 2009; and 74 COYE, 56 YWAR, 50 OCWA and 71 WIWA in 2010. All birds were captured using passive mist netting (Angelier *et al.*, 2010). Fourteen 12 m mist nets were used for 6 h a day, starting 30 min before dawn. Nets were opened every day, weather permitting, with a total of 63, 67 and 62 days between 15 July and 30 September in 2008, 2009 and 2010, respectively. Mist nets were checked every 30 min, and all birds were extracted. All birds were placed in cloth bags and returned to the banding station for processing. Time of capture was estimated relative to time after sunrise; on average, birds were caught 2.32 ± 1.61 h after sunrise (*n* = 838; 5 and 95% quantiles, 0–5.0 h). Birds were blood sampled from the left brachial artery with a 26.5 gauge needle, and blood was collected into heparinized micro-capillary tubes (50–100 μl). All birds were sampled within 30 min of extraction from the mist nets. The mean handling time between extraction from the net and blood sampling was 21.6 ± 10.7 min (*n* = 838; 5 and 95% quantiles, 7.0–41.0 min). After bleeding was complete, birds were banded with aluminum USFW bands, age and sex were identified (Pyle, 1997), morphometric data were recorded (body mass, tarsal length, wing chord length and moult) and feathers were sampled [one tail rectrix (r4) and two primary coverts] before birds were released at the banding station. We included a measure of bird density in our models (see Statistical analyses) by using the total capture rate of all wood warblers (family Parulidae) per net hour for each day we obtained blood sample data (excluding recaptures from the same day). Bird density based on this metric did not vary among years (*F*_2,844_ = 0.028, *P* > 0.70) but did vary with Julian date (*F*_1,844_ = 86.0, *P* < 0.001), indicating that the number of birds increased as migration proceeded.

### Plasma metabolite assays

Blood samples were stored at 4°C for up to 2 h before being centrifuged for 6 min at 10 000***g***. Plasma was collected and stored at −20°C until assayed. Plasma samples were diluted 1:2 with double deionized H_2_O in order to increase the plasma volume available for assay (concentrations of assayed metabolites were diluted linearly). All assays were run in 400 μl, flat-bottom 96-weIl microplates (NUNC, Denmark) and read with a microplate spectrophotometer (Biotec 340EL or Powerwave X 340), as previously described (e.g. Acevado Seaman *et al.*, 2006; Williams *et al.*, 2007). Not all metabolites could be determined for all individuals, because of small plasma volumes; on the basis of previous studies, we prioritized triglyceride and glycerol assays. Free glycerol and total glycerol were assayed via sequential colour end-point assay (Sigma-Aldrich Canada, Oakville, Ontario, Canada), using 5 μl of plasma with 240 and 60 μl of glycerol reagent (A) and triglyceride reagent (B), respectively, with a reading taken at 540 nm after 10 min of incubation at 37°C after the addition of each reagent. Plasma triglyceride concentration (in millimoles per litre) was calculated by subtracting free glycerol from total glycerol. The inter-assay coefficient of variation was 4.1% (*n* = 13 assays), 6.8% (*n* = 15) and 5.2% (*n* = 7) in 2008, 2009 and 2010, respectively. The overall coefficient of variation for all years was 6.7%, but there was some evidence of systematic variation in our standard curve among years. Therefore, in order to compare plasma triglyceride values across years we standardized data for 2008 and 2009 against the slope of the standard curve relating absorbance to concentration for 2010.

β-hydroxybutyrate was measured using a D-3-Hydroxybutyric acid kit (K-HDBA; Megazyme International, Bray, Ireland; Anteau and Afton, 2008; Evans Ogden *et al.*, 2013). Following the kit protocol, 10 μl of diluted plasma was pipetted into a 96-well plate with 272 μl of working solution (double deionized H_2_O, TEA buffer, NAD^+^/INT, Diaphorase suspension), and the plate was read at 2 min to obtain a baseline reading. After adding 2 μl of the active reagent (3-hydroxybutyrate dehydrogenase suspension), subsequent readings were taken every 30 s for 30 min. The rate of change in β-hydroxybutyrate was calculated as the difference in absorbance between 2 and 15 min, and concentrations were determined from a standard curve. The intra-year coefficient of variation for 2008 was 12.2% (*n* = 9), for 2009 15.8% (*n* = 4) and for 2010 5.2% (*n* = 11). The inter-year coefficient of variation for β-hydroxybutyrate assays was 5.8% across all three years (with no evidence of systematic bias among years for β-hydroxybutyrate).

### Stable isotope analysis

Feathers were washed in 2:1 chloroform:methanol solution for 24 h, drained, and then air-dried in a fume hood for an additional 24 h to remove excess solvent. Feather samples (0.3–0.5 mg) were placed in small silver capsules (Elemental Microanalysis, UK) and sent to the University of California Davis Stable Isotope Facility in California, USA. Samples were analysed using a Hekatech HT Oxygen Analyzer interfaced to a PDZ Europa 20:20 isotope ratio mass spectrometer after allowing the samples to equilibrate with laboratory standards for at least 96 h, as described by Wassenaar and Hobson (2003). Feather samples were interspersed with keratin working standards. Preliminary isotope ratios were measured relative to reference gases analysed with each sample and finalized by correcting the values for the entire batch based on the known values of the keratin standards. Stable-hydrogen-isotope (δD) values are expressed as per mil (‰) relative to international standard V-SMOW (Vienna Standard Mean Ocean Water). UC Davis changed their reference standards in 2010 to reduce differences in methodology among isotope facilities. We therefore re-ran a subset of samples collected in 2008 and 2009, and calculated standardized δD values based on the relationship between δD values obtained using the two sets of standards (δD_2010_ = −52.377 + 0.729δD_2008/9_, *r*^2^ = 0.88, *F*_1,20_ = 144.8, *P* < 0.001).

### Statistical analyses

Statistical analyses were all performed in SAS statistical software (version 9.2; SAS Institute). In general, we analysed each species separately as an independent test of each hypothesis, due to concerns about comparing plasma metabolite levels among species (Smith and McWilliams, 2010). Estimated fattening rate is calculated as residual plasma triglyceride levels, controlling for body mass and other covariates (typically, handling time, time of day and Julian date; Guglielmo *et al.*, 2005; Acevado Seaman *et al.*, 2006; Williams *et al.*, 2007). Given that glycerol and β-hydroxybutyrate are measures of fasting, not fattening, we did not expect these metabolites to provide signals of fattening rate in migratory birds, but we analysed these as potential measures of ‘condition’ (Guglielmo *et al.*, 2002, 2005; Landys *et al.*, 2005; Acevado Seaman *et al.*, 2006; Williams *et al.*, 2007).

Our primary interest was to determine whether plasma metabolites varied among years, potentially reflecting variation in water levels (see Results), and whether feather δD values (reflecting the breeding latitude of birds) influenced this relationship. Firstly, we compared variation in plasma triglyceride, glycerol and β-hydroxybutyrate by age and sex classes within each species, pooling all data and controlling for year as a main effect and mass, handling time, time of day, Julian date and bird density as covariates. There was no significant effect of age, sex or the age × sex interaction for plasma triglyceride (*P* > 0.05 in all cases), glycerol (*P* > 0.09 in all cases) or β-hydroxybutyrate (*P* > 0.05 in all cases) in any species. We therefore pooled data by age and sex, and included all individuals of unknown age and sex, in all subsequent analyses.

Secondly, we conducted three multivariate analyses (proc GLM), with plasma triglyceride, glycerol and β-hydroxybutyrate as the dependent variables, year as a main effect, and feather δD values, body mass, handling time, time of day, Julian date and bird density as covariates, for each species separately.

Thirdly, we repeated the multivariate analysis after combining the data from all four species in order to assess whether plasma metabolite levels varied across species, including year, species and year × species as main effects, and the same covariates.

Finally, we conducted three multivariate analyses, with plasma triglyceride, glycerol and β-hydroxybutyrate as the dependent variables, water level as the main effect, species as a factor, and body mass, handling time, time of day, Julian date and bird density as covariates.

For each analysis, we initially ran the full model but then eliminated non-significant terms (*P* > 0.05), and we report statistics for the final reduced model. We calculated water level for the day of capture [water level = elevation of the banding station (438.7 m) minus current reservoir elevation] such that positive values indicate that the site is inundated by water. When the water reached this elevation, the lowest mist-net lines were flooded, so we used this value as the elevation of the banding station. Reservoir water elevation data are collected at Fauquier, Arrow Lake (BC Hydro, unpublished data).

All values are expressed as the lsmean ± SE, unless otherwise noted. Given values for triglyceride, glycerol and β-hydroxybutyrate concentration are residual log_10_ (+1) values corrected for mass, unless otherwise noted.

## Results

### Inter-annual variation in water levels

There was marked annual and seasonal variation in water levels during the study period (Fig. 1). Water levels were highest early in the migration/banding period in all 3 years, but were highest, most similar and above net-elevation early in the season in 2008 and 2010. Conversely, water levels were lowest early in the season and below net level in 2009. In contrast, towards the end of the migratory period water levels were lowest and most similar in 2009 and 2010, and were highest late in the season in 2008 (Fig. 1).

### Variation in body mass

We knew *a priori* that there were species differences in body mass, so we analysed inter-annual variation in body mass for each species separately. There was a significant effect of year on body mass in WIWA (*F*_2,148_ = 3.78, *P* = 0.025) and YWAR (*F*_2,189_ = 3.81, *P* = 0.024), although the patterns differed in the two species. In WIWA, birds were heavier (*P* < 0.01) in 2008 (7.82 ± 0.14 g) compared with 2009 (7.55 ± 0.16 g) and 2010 (7.65 ± 0.11 g). In YWAR, birds were lighter in 2010 (9.08 ± 0.14 g) compared with 2008 (9.36 ± 0.13 g) and 2009 (9.25 ± 0.12 g). In contrast, mean body mass did not differ among years in COYE (*F*_2,273_ = 1.28, *P* > 0.25; mean 10.11 ± 0.13 g) or OCWA (*F*_2,189_ = 0.27, *P* > 0.70; mean, 8.93 ± 0.14 g).

Body mass increased with time of day in OCWA, WIWA and YWAR (*P* < 0.025 in all cases), but not in COYE (*P* > 0.05; controlling for handling time). Body mass increased with Julian date in OCWA (*F*_1,187_ = 15.70, *P* < 0.0001) and WIWA (*F*_1,147_ = 8.02, *P* < 0.01), but not in COYE or YWAR (*P* > 0.05 in both cases). Additionally, there was no effect of year or a time × year interaction for any species (*P* > 0.14 in all cases), and time of day explained only 5.0, 15 and 5% of the total variation in body mass in OCWA, WIWA and YWAR, respectively. There was no effect of year or a Julian date × year interaction for OCWA, WIWA and YWAR (*P* > 0.20). In COYE, there was a significant Julian date × year interaction (*F*_1,269_ = 4.49, *P* < 0.05); mass varied with date in 2008 (*F*_1,94_ = 8.02, *P* < 0.01), but not in 2009 or 2010 (*P* > 0.20).

### Effects of year and feather isotope signature on plasma metabolite values within species

Mean residual plasma metabolite concentrations (log_10_ + 1 lsmeans ± SE) for each species in 2008–2010, controlling for feather δD value, body mass, handling time, time of day, Julian date and bird density (where significant; see below), are given in Table 1. Pooling all data, plasma triglyceride levels were negatively correlated with plasma β-hydroxybutyrate levels (*r* = −0.19, *n* = 621, *P* < 0.001), but plasma triglyceride was not correlated with plasma glycerol (*r* = −0.05, *n* = 832, *P* > 0.10).

**Table 1:** Mean plasma metabolite concentrations (in millimoles per litre) by year and species

Species	Metabolite	Residual plasma metabolite concentration		
		2008	2009	2010
COYE	Triglyceride	1.012 ± 0.018^a^	0.996 ± 0.016^a^	1.036 ± 0.021^a^
OCWA		1.008 ± 0.021^a^	1.025 ± 0.017^a^	1.025 ± 0.023^a^
WIWA		1.127 ± 0.017^a^	1.174 ± 0.020^a^	1.146 ± 0.013^a^
YWAR		0.908 ± 0.021^a^	0.956 ± 0.020^a^	0.964 ± 0.023^a^
COYE	Glycerol	0.589 ± 0.015^a^	0.883 ± 0.013^b^	0.840 ± 0.017^b^
OCWA		0.535 ± 0.020^a^	0.836 ± 0.014^b^	0.824 ± 0.020^b^
WIWA		0.560 ± 0.021^a^	0.908 ± 0.028^b^	0.833 ± 0.018^c^
YWAR		0.516 ± 0.018^a^	0.778 ± 0.016^b^	0.867 ± 0.019^c^
COYE	β-Hydroxybutyrate	1.014 ± 0.020^a^	1.193 ± 0.021^b^	1.003 ± 0.020^a^
OCWA		0.983 ± 0.027^a^	1.266 ± 0.028^b^	1.024 ± 0.027^a^
WIWA		1.008 ± 0.034^a^	1.188 ± 0.042^b^	0.957 ± 0.023^a^
YWAR		1.023 ± 0.023^a^	1.240 ± 0.022^b^	0.958 ± 0.022^c^

Species are coded as follows: COYE, Common Yellowthroat, *Geothlypis trichas*; OCWA, Orange-crowned Warbler, *Oreothlypis celata*; WIWA, Wilson's Warbler, *Cardellina pusilla*; and YWAR, Yellow Warbler, *Setophaga petechia*. Values are log_10_ + 1 lsmeans ± SE, controlling for covariates retained in the model at *P* < 0.05 (feather isotope, body mass, handling time, time of day, Julian date and bird density; see text for details). Values sharing the same letter for rows are not significantly different.

**Figure 2:**
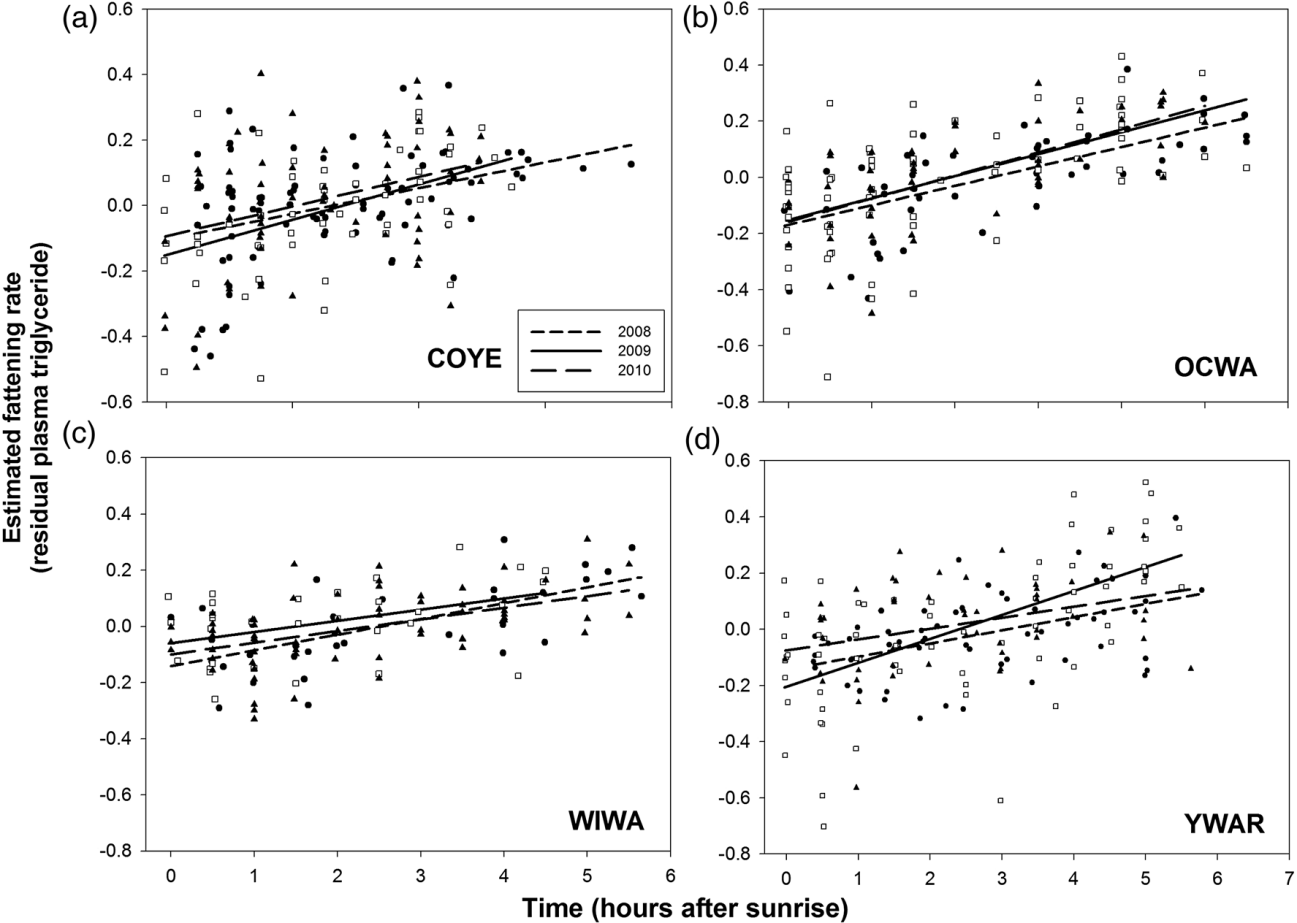
Variation in estimated fattening rate with time after sunrise in four species of migratory passerines. Values are residual log_10_ plasma triglyceride + 1, controlling for feather δD value, body mass, handling time and Julian date. See text for details.

**Figure 3:**
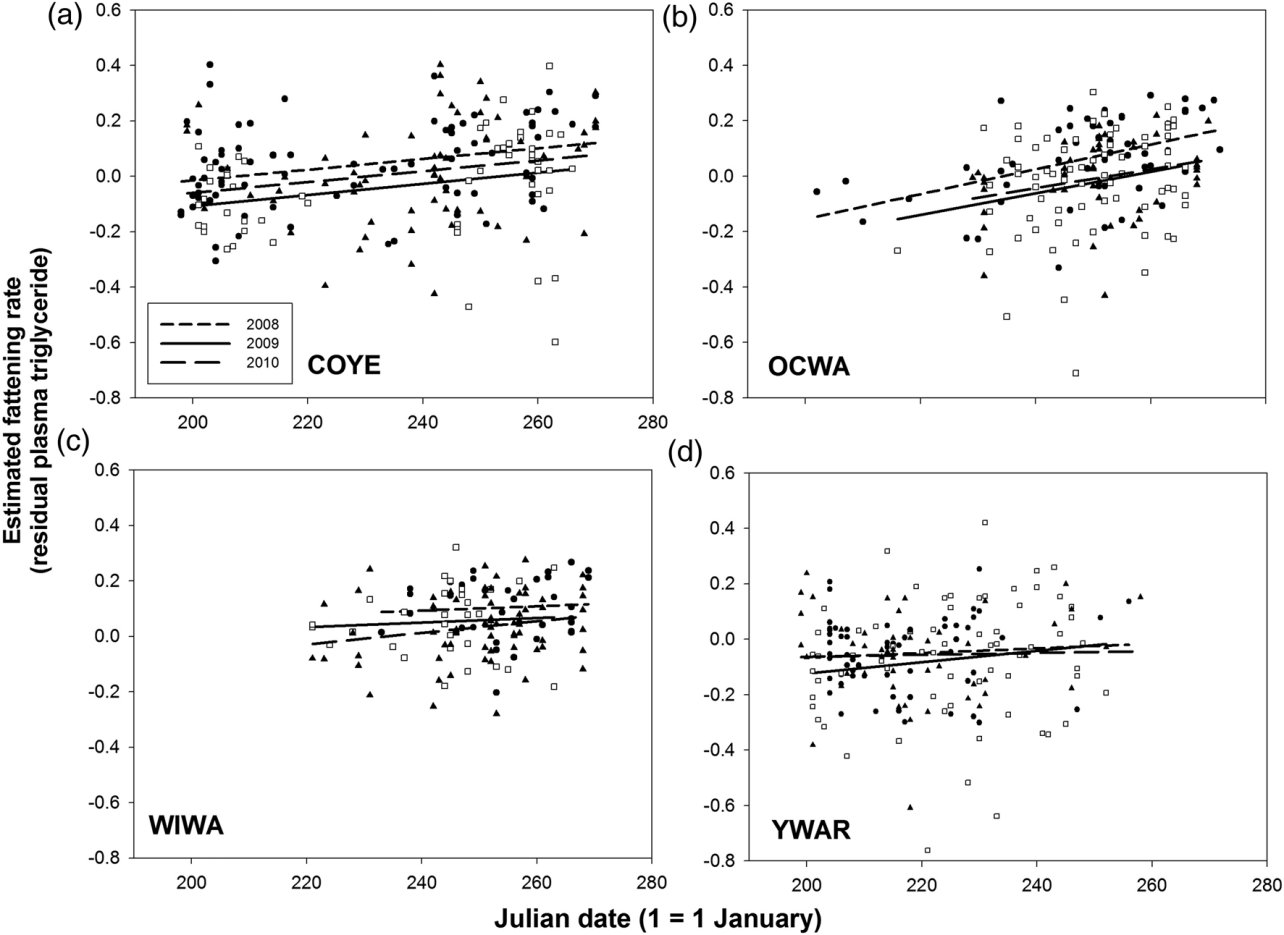
Variation in estimated fattening rate with Julian date (1 = 1 January) in four species of migratory passerines. Values are residual log_10_ plasma triglyceride + 1, controlling for feather δD value, body mass, handling time and time of day. See text for details.

Plasma triglyceride was independent of year in reduced models for all four species (COYE, *F*_2,293_ = 1.15, *P* > 0.30; OCWA, *F*_2,200_ = 0.22, *P* > 0.80; WIWA, *F*_2,148_ = 1.59, *P* > 0.20; and YWAR, *F*_2,198_ = 2.00, *P* > 0.13). Julian date and time of day were the only covariates retained in all models for plasma triglyceride (*P* < 0.001 in all cases); plasma triglyceride increased with time of day (Fig. 2) and with Julian date (Fig. 3) in all four species. There was a significant time of day × year interaction for YWAR (*F*_2,167_ = 3.82, *P* < 0.025; Fig. 2b), but not for the other three species (Fig. 2a–d). Likewise, there was no Julian date × year interaction for any species (*P* > 0.30), i.e. the seasonal increase in plasma triglyceride was similar in all years. Body mass was retained in the model for WIWA only (*F*_1,129_ = 5.03, *P* < 0.05). Feather δD value, handling time and bird density were not significant in any models (*P* > 0.15) for any species. The lack of effect of feather δD was not due to lack of variation in isotope signatures, which was in fact large, i.e. mean δD for COYE, −154.7 (range −191.1 to −128.8, *n* = 217), OCWA −162.9 (range −190.3 to −130.4, *n* = 179), WIWA, −143.9 (range −177.2 to −107.9, *n* = 135) and YWAR, −158.3 (range −189.9 to −141.2, *n* = 190). Feather δD varied with Julian date in all four species (*P* < 0.025). However, the slope and strength of the relationship varied among species; feather δD increased with date in COYE (*b* = 0.059 ± 0.025, *r*^2^ = 0.025) and, more markedly, in WIWA (*b* = 0.509 ± 0.106, *r*^2^ = 0.148), but decreased with date in OCWA (*b* = −0.154 ± 0.059, *r*^2^ = 0.037) and YWAR (*b* = −0.095 ± 0.037, *r*^2^ = 0.033).

For both plasma glycerol and β-hydroxybutyrate, there was a highly significant effect of year (*P* < 0.001 in all cases; Table 1); however, the pattern of inter-annual variation was not consistent for these two metabolites. Plasma glycerol levels were lower in 2008 compared with 2009 and 2010 (*P* < 0.001) in all four species, whereas plasma β-hydroxybutyrate was higher in 2009 compared with both 2008 and 2010 (*P* < 0.001; Table 1). There were few consistent relationships with other covariates. Bird density was retained in the model for plasma glycerol for COYE (*P* < 0.01) and OCWA (*P* < 0.05), but not for WIWA or YWAR, or for β-hydroxybutyrate in any species. Time of day and handling time were significant for plasma glycerol in YWAR only (*P* < 0.05 and *P* < 0.001, respectively), and time of day was significant for plasma β-hydroxybutyrate in WIWA only (*P* < 0.001); Julian day was significant in the model for β-hydroxybutyrate in OCWA only (*P* < 0.001).

### Differences in estimated fattening rate among species

Estimated fattening rate varied among species (*F*_3,848_ = 22.03, *P* < 0.001), being higher in WIWA compared with all other species (*P* < 0.001; Table 1). The only other terms retained in the model were time of day (*P* < 0.001) and Julian date (*P* < 0.001). There was also an effect of species on plasma glycerol (*F*_3,669_ = 11.82, *P* < 0.001), but here glycerol was higher in COYE than in all other species (*P* < 0.025 in all cases; Table 1; year, feather δD value, body mass, time of day, Julian date and bird density were retained in the model, *P* < 0.05). In contrast, there was no difference in plasma β-hydroxybutyrate levels among species (*F*_3,619_ = 1.26, *P* > 0.25; year *P* < 0.001 and time of day *P* < 0.01).

### Effect of daily variation in water levels on plasma metabolite levels

**Figure 4:**
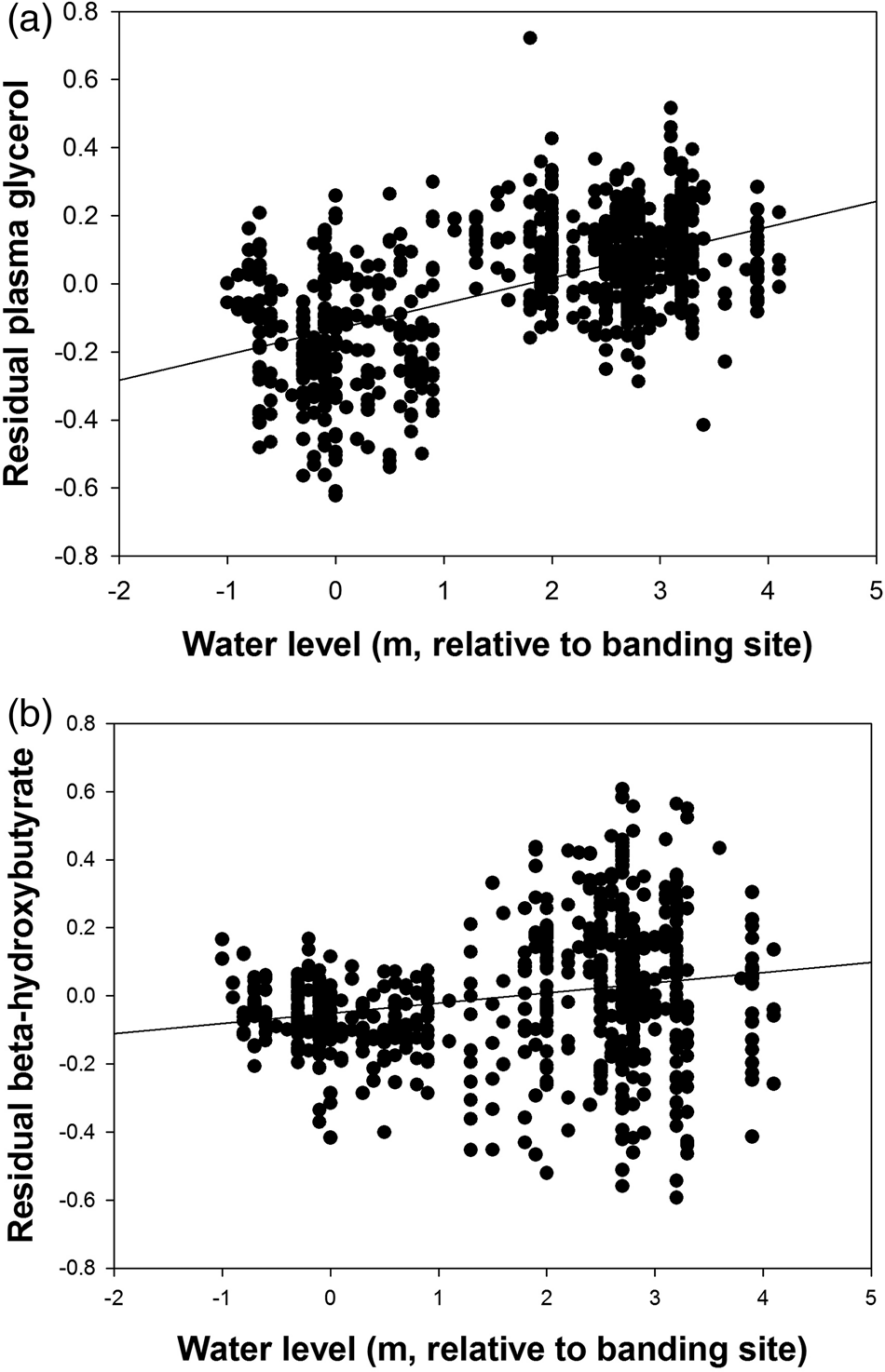
Variation in plasma glycerol (**a**) and plasma β-hydroxybutyrate (**b**) in relationship to water levels (defined in Fig. 1). Values are residual metabolite levels from a model including species, body mass, handling time, time of day and Julian date.

Plasma triglyceride was independent of daily water level (*F*_1,848_ = 1.16, *P* > 0.25) and there was no species × water level interaction (*P* > 0.70). There was an effect of species (*P* < 0.001; confirming the species difference reported above). In contrast, plasma glycerol varied with water level (*F*_1,785_ = 601.4, *P* < 0.001) and species (*F*_3,785_ = 11.58, *P* < 0.001; as above), but there was no species × water level interaction (*P* > 0.60). Finally, for β-hydroxybutyrate there was an effect of water level (*F*_1,620_ = 26.5, *P* < 0.001), no effect of species (*P* > 0.60; as above), but there was a species × water level interaction (*F*_3,620_ = 2.99, *P* < 0.05). Water level affected β-hydroxybutyrate in COYE, OCWA and YWAR (*P* < 0.01 in all cases), but not in WIWA (*P* > 0.90). In both cases, residual plasma metabolites levels increased with decreasing water levels (i.e. with drier conditions), and this relationship was stronger for glycerol (*F*_1,829_ = 360.3, *P* < 0.001, *r*^2^ = 0.30; Fig. 4a) than for β-hydroxybutyrate (excluding WIWA, *F*_1,473_ = 21.3, *P* < 0.001, *r*^2^ = 0.04; Fig. 4b).

## Discussion

Our main goal in this study was to determine the following: (i) whether plasma triglyceride levels, as a measure of estimated fattening rate, varied among years with very different patterns of variation in water levels, or with daily variation in water levels, in reservoir-impacted riparian habitat; and (ii) whether feather δD values [reflecting variation in the geographical origin (breeding latitude) of migratory birds] influenced this relationship. We confirmed that: (i) plasma triglyceride varied systematically with time of day and Julian date; (ii) estimated fattening rate varied among species; but (iii) estimated fattening rate did not vary with age or sex, all of which confirms results of previous studies (see below) and supports the idea that plasma triglyceride does provide an index of fattening rate. However, we found no inter-annual variation in estimated fattening rate, even though there were marked differences in water levels among years. Likewise, there was no relationship between daily water levels and estimated fattening rate. Furthermore, data on feather δD values did not add to the explanatory power of models in any of our analyses. Finally, we did find inter-annual variation in plasma glycerol and β-hydroxybutyrate levels and significant, though weak, relationships between these metabolites and water level (higher metabolite levels when drier).

Our study site was selected as a migration monitoring station by Parks Canada in 1998. It represents one of the few remaining pieces of riparian habitat within the Canadian portion of the Columbia River Basin and is known to be used by migratory birds in the autumn. For some neotropical migrants that do not breed at the study site (e.g. Wilson's Warbler and Orange-crowned Warblers), capture rates are >20 times higher during the peak of the migration period (late August) than at the end of July (Green *et al.*, 2011). For species that do breed at the site (e.g. Common Yellowthroats), capture rates drop slightly between the end of July and the first week of August, but then increase 4-fold during the peak of the migration (83% of samples were obtained during peak migration in August/early September). Fattening rates at this site were estimated to range from 0.32% lean body mass h^−1^ for American Redstarts and 0.98% lean body mass h^−1^ for Wilson's Warblers (Green *et al.*, 2011), similar to estimates from 15 other migration monitoring sites in Canada (average for 14 species = 0.53% lean body mass h^−1^) and above the estimated 0.24–0.26% lean body mass h^−1^ required to cover overnight energy use (Dunn, 2002). Overall, our mean plasma triglyceride levels (∼1 mmol l^−1^) were within the range reported for refuelling migratory birds in other studies (e.g. 0.8–1.8 mmol l^−1^, Guglielmo *et al.*, 2005; Seewagen *et al.*, 2011). Thus, we are confident that the majority of birds we sampled are true migrants, and that birds fatten at this site at rates that exceed those needed to cover overnight energy use and to accumulate fuel for migration.

In our study, time of day and Julian date were the variables most strongly and most consistently related to variation in plasma triglyceride levels, across all four species. Numerous studies using plasma metabolite analysis have reported an increase in estimated fattening rate with time since dawn, consistent with an overnight fast and resumption of feeding at dawn (Guglielmo *et al.*, 2002, 2005; Williams *et al.*, 2007; Evans Ogden *et al.*, 2013). Fewer studies have investigated seasonal changes, but in Western Sandpipers the estimated fattening rate increased with sampling latitude, i.e. as spring migration proceeded (Williams *et al.*, 2007). At the same site in Revelstoke where we conducted our study, Green *et al.* (2011) found no change in mass gain across weeks of the migration period using capture mass regressed against capture time, suggesting that plasma metabolites provide a more sensitive assay of seasonal variation in fattening rate (see also Williams *et al.*, 2007).

Several factors had little effect or no consistent effect on plasma triglyceride levels. Body mass was only weakly (positively) related to plasma triglyceride levels in two of four species in our study, and the pattern of variation in body mass among years was not consistent between or within species. Individuals tended to be heavier in 2008 than in subsequent years in WIWA and YWAR, but this did not hold for other species. Likewise, in four species of migrants, fat score and body mass were not related to time of day or date and did not vary consistently with altitudinal differences in estimated fattening rate based on plasma triglyceride analysis (Evans Ogden *et al.*, 2013). These studies support the idea that body mass and other ‘static’ measures (e.g. fat score) may not be as sensitive to, or informative of, variation in ‘condition’ or habitat quality in migratory passerines (Moore and Kerlinger, 1987; Guglielmo *et al.*, 2002; Jenni-Eiermann *et al.*, 2002; Evans Ogden *et al.*, 2013). We also found no detectable effect of age or sex on estimated fattening rate during autumn migration. This is consistent with previous studies that found no age or sex differences either using alternative methods of estimation of fattening rate [fat score, Yong *et al.*, 1998; Evans Ogden *et al.*, 2013; mass × time regression, Jones *et al.*, 2002 (for most species); Green *et al.*, 2011] or plasma metabolite analysis (e.g. Guglielmo *et al.*, 2002; Acevado Seaman *et al.*, 2006; Evans Ogden *et al.*, 2013). From a sampling or monitoring perspective, this is an important result because it means that data can be pooled by age and sex, thereby maximizing statistical power to detect main effects of year or habitat, which are likely to be of primary conservation-related interest. Finally, even though variation in water levels can markedly reduce the available area of riparian habitat (by 40–90% at our study site, Green *et al.*, 2011), which would be expected to increase the densities of, and perhaps competition between, foraging birds, we found no evidence that bird density affected the estimated fattening rate based on plasma triglyceride levels. Green *et al.* (2011) found no evidence that water level influenced bird density (assessed using capture rate of the five warbler species in that paper) or that density had an effect on rates of mass gain calculated using mass × time regression (but see Moore and Yong, 1991).

Geographical origin is known to influence the timing of autumn migration in Wood Warblers (Kelly, 2006) and might therefore be expected to influence fattening rates. Kelly (2006) demonstrated that Common Yellowthroats and Orange-crowned Warblers from the southern portion of their range pass through New Mexico before northern conspecifics in the autumn. In contrast, Yellow Warblers and Wilson's Warblers from northern breeding sites precede southern conspecifics. Geographical origins would influence fattening rates if local birds had not entered the pre-migratory phase while migrating northern conspecifics exhibited periods of hyperphagia and rapid fat deposition (Berthold, 2001), or if birds from different geographical origins employed different migratory strategies or migration routes, requiring different energy reserves (e.g. Fitzgerald and Taylor, 2006; Coiffait *et al.*, 2011). Feather δD did not enter into any of our models as an explanatory variable for variation in plasma triglyceride levels, suggesting that geographical origin does not influence fattening rates of any species in this study. Although feather δD did vary with Julian date, these relationships were generally weak and differed among species, whereas the pattern of variation in triglyceride and date was consistent among all four species, again suggesting the geographical origin is not a major driver of fattening rate. This suggests that the majority of birds had entered the pre-migratory phase prior to capture, and that birds captured at a single location, and at the same time, may face similar challenges and thus employ similar migratory strategies.

Controlling for other sources of variation in plasma triglyceride levels, we found significantly higher mean estimated fattening rates (residual triglyceride) in WIWA compared with the other species in each of the 3 years. Smith and McWilliams (2010) urge caution when making comparisons of estimated fattening rate among species. However, in our study all species were sampled at a single site over multiple years, all have mainly insectivorous diets, and all species showed similar trends between plasma triglyceride, time of day and date. Green *et al.* (2011) used regression analysis of body mass by capture time to estimate daily mass gain of five warbler species at the same study site in Revelstoke, British Columbia and also found that WIWA had the highest fattening rate among species. Our data therefore suggest that, when used carefully, plasma metabolite levels could be used to investigate species-level variation in fattening rates.

In contrast to our data on plasma triglyceride, we found significant inter-annual variation in mean plasma glycerol and β-hydroxybutyrate levels, and both these metabolites were related to variation in daily water levels, with higher residual metabolite levels in drier conditions, and with glycerol showing stronger relationships. Elevated plasma levels of glycerol and β-hydroxybutyrate are typically considered to be measures of fasting and fat catabolism. However, this is usually associated with extreme fasting (e.g. in large-bodied birds, Cherel *et al.*, 1988) or following endurance flights where birds are sampled immediately after landing (Jenni–Eiermann and Jenni, 1991). Other studies of birds at migratory stopovers, where most birds will be feeding, fattening and not fasting, have shown that plasma glycerol and β-hydroxybutyrate can be highly variable and less informative for assessing fattening rate (Guglielmo *et al.*, 2002, 2005; Landys *et al.*, 2005; Acevado Seaman *et al.*, 2006; Williams *et al.*, 2007; Smith and McWilliams, 2010). We did find the predicted inverse relationship between plasma triglyceride and β-hydroxybutyrate (e.g. Cerasale and Guglielmo, 2006; Anteau and Afton, 2008), although this relationship was weak (*r*^2^ = 0.04). Furthermore, we did not find consistent relationships between plasma β-hydroxybutyrate or glycerol and time of day or date factors, which should predict fattening rate (Guglielmo *et al.*, 2002, 2005; Williams *et al.*, 2007; Evans Ogden *et al.*, 2013). This suggests that β-hydroxybutyrate does not simply reflect the inverse of fattening rate (i.e. fasting) in our study, where most birds were likely to be actively feeding at capture. Rather, these metabolites might be varying for some other reason. Several studies have suggested that variation in β-hydroxybutyrate can reflect differences in dietary composition or dietary quality, confounding a simple interpretation of these metabolites (Guglielmo *et al.*, 2005; Cerasale and Guglielmo, 2006; Smith *et al.*, 2007). Smith *et al.* (2007) showed that during feeding, White-throated Sparrows (*Zonotrichia albicollis*; a migratory songbird) fed a high-protein insect diet had higher plasma β-hydroxybutyrate concentrations than birds fed a low-protein diet, even though diet did not affect short-term mass change. However, these studies did not look at the effect of diet on glycerol. Guglielmo *et al.* (2005) reported a U–shaped pattern for plasma glycerol in migratory passerines sampled at a stopover site, and they suggested this might reflect increased glycerol production during lipolysis, but also rapid fatty acid uptake by adipose tissue and muscle when birds have high fattening rates. Cerasale and Guglielmo (2006) also cautioned against the use of glycerol in studies involving plasma metabolite profiles because of its dual role in lipolysis and fat deposition, unless all measurements are in a range where the relationship between triglyceride and glycerol is linear. Clearly, this is not the case in our study, and the potential complexity of interpreting glycerol values is at least supported by the fact that we did not find an inverse relationship between plasma triglyceride and glycerol, as would be predicted if elevated glycerol reflects only high rates of lipid mobilization. It is possible, therefore, that the positive relationship between β-hydroxybutyrate, and perhaps glycerol, reflects a difference in dietary composition or dietary quality associated with lower water levels (drier conditions), but this does not translate into differences in fattening rate.

Finally, in relationship to the primary goal of our study, we found no evidence that estimated fattening rate varied among years with very different patterns of variation in water levels, or with daily variation in water levels, in this reservoir-impacted riparian habitat. Previous studies have shown that there can be consistent differences in plasma triglyceride levels between different habitats, including habitats at migratory stopover sites (Acevado Seaman *et al.*, 2006; Williams *et al.*, 2007; Seewagen *et al.*, 2010; Smith and McWilliams, 2010; but see Thomas and Swanson, 2014). Guglielmo *et al.* (2005) showed that an independent measure of stopover habitat quality (measured by mass gain) was correlated with plasma triglyceride levels. We do not think that our conclusion is influenced by non-random sampling of birds at first capture that might have low fattening rates associated with ‘settling’ at this site. Settling costs have mainly been documented in long-distance migrants that land after crossing major migratory barriers (e.g. deserts, oceans), whereas our study species are primarily short-hop or leap-frog migrants, which might move more-or-less continuously through feeding/refuelling habitat. Low recapture rates at our site (∼23% more than 1 day after initial capture) suggest that the majority of birds arrive, fatten and move on rapidly (see Green *et al.*, 2011). Thus, our study supports the conclusion of Green *et al.* (2011) that, at present, although hydroelectric dam operations influence water levels in the Arrows Lake Reservoir and adjacent riparian habitats, this does not significantly impact fattening rates of migratory passerines using these habitats (at least within the range of water levels generated by reservoir operation in our study years; clearly, more extreme flooding would cause a greater reduction in the area of riparian habitat, Green *et al.*, 2011). The remnant riparian habitat along the Columbia River in British Columbia, therefore, currently provides warblers with stopover habitat that allows them to gain body mass and fuel southward migration, but this highlights the need to conserve this remaining habitat.
